# Gastric Function in Children with Oesophageal Atresia and Tracheoesophageal Fistula

**DOI:** 10.3389/fped.2017.00076

**Published:** 2017-04-12

**Authors:** Gilles Duvoisin, Usha Krishnan

**Affiliations:** ^1^Department of Paediatric Gastroenterology, Sydney Children’s Hospital, Randwick, NSW, Australia; ^2^Department of Paediatrics, Lausanne University Hospital, University of Lausanne, Lausanne, Switzerland; ^3^School of Women’s and Children’s Health, University of New South Wales, Kensington, NSW, Australia

**Keywords:** oesophageal atresia/tracheo-oesophageal fistula, children and adolescents, gastric motility, gastric emptying, electrogastrography, octanoic breath test, dumping syndrome, prokinetic

## Abstract

Oesophageal atresia and tracheoesophageal fistula (OA–TOF) are a multifaceted condition which affects patients throughout their lives. Even though it is one of the most common gastrointestinal malformations, most of the current studies focus on gastro-oesophageal reflux disease, anastomotic strictures, and feeding difficulties. However, there is increasing evidence that a proportion of patients with OA–TOF also have abnormal gastric function. This review aims to provide a comprehensive understanding of studies of gastric function in patients with OA–TOF. The etiology of this abnormality has been hypothesized to be congenital and/or acquired. Several modalities are currently available for the investigation of gastric function, each of them trying to answer specific clinical questions. This review summarizes the studies that have looked at gastric function in the OA–TOF cohort with gastric emptying studies (gastric emptying scintigraphy and ^13^C octanoic breath test), gastric manometry, electrogastrography, and oral glucose tolerance test. However, these modalities are limited due to poor age-specific normative values and heterogeneous methodologies used. The evaluation of symptoms in this cohort is crucial, modalities for abnormal gastric function are also described. With appropriate investigations and symptoms questionnaires, treatment strategies can be implemented to correct abnormal gastric function and thereby improve the outcomes and quality of life of patients with OA–TOF. This review highlights the need for large international multicentre collaborative studies and high-quality prospective randomized controlled trials to improve our understanding of gastric function in this cohort.

## Introduction

Oesophageal atresia (OA) and tracheoesophageal fistula (TOF) are defined as an interruption of the continuity of the oesophageal lumen, which can be associated with or without the presence of a TOF. The abnormal communication can occur between the proximal and/or the distal oesophagus and the trachea. OA–TOF is known to be one of the most common malformations in the gastrointestinal tract. Regarding its incidence, OA–TOF has been reported in approximately 1 in 3,500 births (1).

Recently, there have been significant improvements in the care of patients with OA–TOF with the involvement of multidisciplinary teams including the surgeon, gastroenterologist, otorhinolaryngologist, pulmonologist, nutritionist, speech pathologist, and psychologist. This team approach to the care along with improvements in the initial neonatal management (intensive care, anesthesia, ventilatory, and nutritional support) in association with the primary surgical repair, has resulted in a significant reduction of the mortality with a current survival rate as high as 95% in centers with the best neonatal care (2). Most of the patients who do not survive the first months of life often have severe concomitant malformations. Despite this reduction in the mortality rate, children with OA–TOF can demonstrate a significant amount of long-term complications and suffer from lifelong morbidity due to complications, such as gastroesophageal reflux disease (GORD), oesophageal dysmotility, Barretts oesophagus, anastomotic strictures, and feeding difficulties.

The aim of the recent consensus guidelines on the management of gastrointestinal complications in these patients by NASPGHAN and ESPGHAN was to improve patient-related outcomes and the quality of life of patients by reducing the morbidity from gastrointestinal complications in this cohort (3).

While much is known about the abnormal oesophageal function and poor motility in OA–TOF patients (4, 5), little is known about gastric function in OA–TOF patients. It has been postulated that abnormalities in gastric function may contribute to high prevalence of gastrointestinal complications, such as GORD and feeding difficulties in this cohort. This review will discuss the etiology of abnormal gastric function, investigations of gastric function, symptoms that could be secondary to abnormal gastric functions, and finally potential treatment strategies for abnormal gastric function in this cohort.

## Pathophysiology

The normal gastric motor function is a complex sequence of events. All of them are controlled by an extrinsic nerve supply (brain and spinal cord), myenteric plexus within the wall of the stomach, and the result of local transmitters (amines and peptides), that modulate the excitability of the smooth muscle of the stomach.

### Autonomic Nervous System

The parasympathetic pathway is transmitted to the stomach *via* the vagus nerves. Qi et al. (6) have described congenital abnormalities in the course and branching of the vagus nerves in a rat model with OA–TOF. The left vagus nerve consistently followed an abnormal path below the aortic arch, which could potentially increase the risk of damage to the vagus at time of initial repair of the OA–TOF. It has also been postulated that complications, such as postoperative infections, anastomotic strictures, leaks, and tension and ischemia at anastomotic site, could have a further adverse effect on the integrity of the vagi and thereby affect gastric function.

### Enteric Nervous System

Focusing on the enteric nervous system, Nakazato et al. (7) documented an abnormal development of the myenteric plexus (Auerbach plexus) in the oesophagus as well as in the stomach in a small series of five patients. Most of the gastric biopsy specimens of patients showed significantly larger ganglia and thicker inter-ganglionic fibers than normal, and the network was also looser than normal. Interestingly, in an animal OA–TOF model, anomalies of the myenteric plexus of the oesophagus in OA–TOF rats were also reported by Qi et al. (8). They describe a reduction of the number of cell bodies within the ganglia and a decrease of the density of ganglia and nerve fibers. These abnormalities in the enteric nervous system could also have an adverse effect on gastric function.

### Gastric Smooth Muscles

There are a paucity of studies investigating the role of the gastric smooth muscles contractility. Tugay et al. (9) reported physiologic changes of the smooth muscle of stomach in rat fetuses with OA–TOF in comparison with controls. This was investigated *via* both receptor-dependent (carbachol, serotonin, isoproterenol) and receptor-independent agonist (KCl, papaverine) mechanisms. The results showed an inadequate gastric muscular contraction in both mechanisms. However, this was an *in vitro* animal study which limits its extrapolation to *in vivo* gastric function.

In summary, there is limited evidence that congenital and acquired abnormalities of the autonomic nervous system (extrinsic), enteric nervous system (intrinsic), and potentially even the gastric smooth muscle could result in abnormal gastric function in OA–TOF patients. However, as a lot this evidence was either from animal studies or small case series, further corroboration needs to be done in larger cohorts’ studies on patients with OA–TOF.

## Clinical Symptoms of Abnormal Gastric Function

Most of the questionnaires currently available, such as the gastroparesis cardinal symptom index, which is itself a subset of the PAGI-SYM (patient assessment of upper gastrointestinal disorders-symptoms) are not validated in a pediatric population (10–12). No questionnaire exists for a proxy-report. To assess gastrointestinal symptoms in children, the Rome Foundation establishes questionnaires especially for gastrointestinal functional disorders (13). The PedsQL Gastrointestinal Symptoms Module was established and validated to use several scales to report the quality of life of children, such as symptoms scale, worry scale, medication scale, and communication scale (14). It can assess functional gastrointestinal disease or organic disease (15). The PedsQL currently has subsections which relate to gastric dysfunction. Questions on stomach pain and discomfort after eating, limitation of the child’s ability to eat certain foods, early satiety, nausea, vomiting, sensation of “bloating,” and abdominal distension, all give information on the presence of gastric dysfunction in the PedsQL. However, the difficulty in gastric dysfunction is that the symptoms are non-specific, and especially in an OA–TOF patients, in whom GORD, eosinophilic oesophagitis, and oesophageal dysmotility may result in similar symptoms.

## Investigations of Gastric Function

Diagnosing abnormal gastric function in children, including those with OA–TOF, is challenging as several modalities are available, and each of them may provide a different physiologic answer.

Gastric function can be studied by evaluating gastric emptying (GE), gastric smooth muscle function, and gastric myoelectrical activity. GE can be assessed by scintigraphy or octanoic acid breath test. Gastric smooth muscle function can be studied by gastric manometry, and surface electrogastrography (EGG) can evaluate the gastric myoelectrical activity. We will also describe the oral glucose tolerance test (OGTT) that can be used to determine the presence of dumping syndrome.

### GE—Gastric Emptying Scintigraphy (GES)

Gastric emptying scintigraphy is an objective physiologic non-invasive test that provides a quantitative measurement of the GE. Even though it represents a standard method to measure GE, GES has several limitations such as the standardization of the meals used and the duration of the imaging. In 2008, the Society of Nuclear Medicine, American Gastroenterological Association, and Neurogastroenterology and Motility Society (16) described in a consensus statement the standardized measurement of GE in adults, mainly based on the work of Tougas et al. (17). In this study, the recommended low-fat meal consists of white bread, egg-white, jam, and water, and they recommend that the images be taken at 0, 1, 2, and 4 h after its ingestion. In 2015, Wong et al. (18) retrospectively examined this protocol in a pediatric population. They documented the difficulty for some children to finish the standard meal and the importance of documenting anthropometric factors (lower weight, height, and body surface area) and age which could influence the results of the GES.

Jolley et al. (19) were the first to investigate GE in 25 children with repaired OA–TOF. Only 20 of the 25 had GES. The aim of the study was not only to evaluate GE but also to see whether there was an association between GORD diagnosed *via* pH monitoring and gastroesophageal scintiscan and delay GE documented in GES. GE was slower in OA–TOF patients who had documented GORD on gastroesophageal scintiscan (*p* < 0.005). The main limitation of this study is that they used a liquid meal to assess GE, and the analysis was limited to the GE at 30 min (17); also, no definition of delayed GE was given. Furthermore, gastroesophageal scintiscan is not recommended for routine evaluation of pediatric patients with suspected GORD (20). This study also showed that slow GE was present in a subset of patients with GORD, diagnosed via a reflux score which was determined by pH monitoring. However, a reflux score, rather than an acid reflux index, was correlated with GE. The only factor associated with higher incidence of significant GORD and slow GE was an excessive tension at the anastomotic site (*p* < 0.005), potentially due to a decrease of the intra-abdominal oesophageal length and alteration of the configuration of the gastroesophageal junction.

In addition to this study, for the first time, Montgomery et al. evaluated 11 OA–TOF patients (age 5–10 years, median 7.5 years) and 10 healthy controls with a GE using a solid meal and a symptom questionnaire (21). All the GE parameters measured, such as the half-emptying time, lag phase (time-point when 90% of the marker remained in the stomach), and corrected half-emptying time (half-emptying time minus the lag phase), were significantly prolonged in OA–TOF patients when compared to controls. Also, in OA–TOF patients, the retention values at 60 and 90 min were increased and the emptying rates (percentage of emptying per hour) were reduced in OA–TOF patients. Twenty-seven percent of OA–TOF children (3 over 11) had a delayed GE (e.g., retention values at 60 and 90 min above 2 SDs). Regarding their clinical findings, there was no statistical difference in the GE studies in patients with or without symptoms (abdominal complaints and reflux symptoms).

In summary, although GES can be used to assess GE, here is, however, a dearth of data on normal values in children due to its low but non-negligible radiation risk. There is also a lack of standardization of the type (liquid vs. solid and caloric content) of meals used and the duration of the study. None of the studies mentioned above on OA–TOF patients followed the protocol recommended by Abell et al. (16) and Tougas et al. (17).

### GE—13 C Octanoic Acid Breath Test (OBT)

Due to the drawbacks associated with the scintigraphy methods (mentioned previously), alternative techniques of assessing GE have been recently developed. ^13^C octanoic acid breath test OBT is a radiation-free method used to determine the GE rate of solid (22) and liquid meals in children or adults. This test assumes a normal absorption of the octanoic acid in the small bowel and normal lung function to determine the ratio of ^13^CO_2_/^12^CO_2_ in exhaled breath. Three mains parameters are calculated: the GE half-time (GE *t*1/2), the lag phase (T lag), and the gastric emptying coefficient (GEC). Most of the studies have found a significant linear correlation with GE as determined by scintigraphy with respect to the GE half-time time and the lag phase (22–25). The GEC is specific for OBT.

Van Wijk et al. (26) were the first to combine multichannel intraluminal impedance–pH monitoring as a measure of GORD, oesophageal manometry as a measure of oesophageal motility and function, and GE *via* OBT as a measure of GE to evaluate the mechanisms underlying GORD in this cohort. They recruited 10 children and 10 adults with OA–TOF. Among them, seven infants and nine adult patients were assessed by an OBT with a liquid and solid meal, respectively. Delayed GE (>90th percentile of age-, meal-, and sex-appropriate normal values) was found in 57.1% of infants (four infants over seven) and 22.2% of adults (two adults over nine). When GE half-life was compared to oesophageal motility or bolus clearance, no associations were found (*R* = −0.48, *p* = 0.32 and *R* = −0.56, *p* = 0.23, respectively). However, the normal values used to assess the delayed GE were not available in the manuscript or referenced as published data. In addition, the choice of defining an abnormal GE half-life being above the 90th percentile may overrepresent the gastric dysfunction.

### Gastric Smooth Muscle Function—Gastric Manometry

Conventional manometry and more recently high-resolution manometry have added a new method to assess gastric motility (27, 28). Conventional gastric manometry is not a commonly used method to study gastric motility, and high-resolution manometry needs further investigation to understand its role and compare its finding with other clinical tests. There has been only one study so far which used conventional manometry to study gastric function in OA–TOF patients. Eleven OA–TOF patients, aged from 13 to 23 years, were recruited by Romeo et al. to evaluate their gastric function *via* GES and gastric manometry (29). Like Montgomery et al., they used a solid meal to assess the GE, in contrast to Jolley et al. who chose a liquid meal. Delayed GE was defined as a t½ more than 90 min and was seen in 36% of the patients (4 over 11). Two of them were symptomatic of GORD, and two remaining patients were asymptomatic. All four presented with altered gastric motility at manometry. Also, 45% of patients demonstrated abnormal gastric peristaltic activity and antral hypo motility on manometric testing. This involved an increased duration of the third phase of the interdigestive cycle, reduction of the frequency and reduction of the amplitude of the peristaltic waves. However, like the previous studies, there was poor correlation between manometry results and symptoms, and abnormal gastric motility was also seen in 20% of asymptomatic patients. This work was the first to study gastric function in adult OA–TOF patients and showed that although gastric motility disorder can still be present in adulthood, it may not always be responsible for the GI symptoms. The authors felt that evaluation of gastric function may be useful in dyspeptic older OA–TOF patients.

### Myoelectrical Activity—EGG

Electrogastrography is a non-invasive method for the measurement of gastric myoelectrical activity using cutaneous electrodes (30). There is increasing evidence of its validity since the 1990s. EGG chiefly provides information on myoelectrical rhythm and amplitude/power of the stomach. If the recording of the electrogastrogram follows an adequate preparation of the skin and electrode placement, it is an accurate measurement of gastric slow waves (31). There are currently no recommendations regarding a standard meal for EGG. However, the meal composition is important as solid, liquid, or containing a high percentage of fat may result in different postprandial EGG responses (32, 33). Established EGG parameters are derived from the spectral analysis, and used to classify the result of the EGG. The parameters looked at include dominant frequency (frequency appearing with peak power value of spectra), dominant power (the power observed at the dominant frequency), power ratio (ratio between the power in the postprandial period to the fasting period), percentage of normal gastric slow waves [the frequency of normal slow waves is between 2 and 4 cycles per minute (cpm)]. The EGG is described as bradygastria if the dominant frequency is less than 2 cpm and tachygastria if it is higher than 4 cpm and less than 9 cpm. Arrhythmia is defined if no dominant power is documented. Finally, bradygastria, tachygastria, or arrhythmia defines the presence of dysrhythmia. Although dysrhythmia can be found in healthy controls, normogastria (slow waves between 2 and 4 cpm) should represent more the 70% in healthy controls (34). Several studies described a variation in their normative values, increasing the difficulty to compare them.

Cheng et al. (35) were among the first to assess children with OA–TOF *via* EGG. Their study looked at 18 OA–TOF patients and 10 healthy controls, with a mean age of 2.3 years (2 weeks to 12 years) and 2.1 years (1 month to 10 years), respectively. First, the dominant frequency did not differ significantly between the two groups. Even though, the instability coefficient (SD divided by the mean value of frequency) is the best-established parameter to describe the variation of the regularity of the slow waves, the distribution of frequency was used in the study. OA–TOF patients had a significantly wider distribution of frequency than the controls. The authors postulated that this was secondary to either the gastric pacemaker cells not firing at a regular rhythm or due to abnormalities in intrinsic nerves which modulate smooth muscle cells resulting in poor electromechanical coupling and abnormal gastric contraction. Interestingly, none of the four patients with abnormal EGG (two patients with bradygastria and the two patients with tachygastria) were symptomatic. However, no validated questionnaire was used to determine the presence or absence of reflux symptoms.

Yagi et al. (36) evaluated the gastric function using EGG in 13 OA–TOF children, aged from 1 to 17 years old (mean 7.6 years), and compared them with five controls. EEG anomalies were only reported in OA–TOF, and they were present in 38% of them (5 over 13). Yagi et al. defined dysrhythmia when the SD of peak spectral frequencies was larger than 1.3. Even though this definition varies in the literature, it is significant that only OA–TOF patients (38.4%) in this study had dysrhythmia (two only in the postprandial period and three in the fasting and postprandial period). In addition, the power ratio of the controls was significantly higher than that of the OA–TOF patients (7.6 ± 9.0 vs. 2.6 ± 1.7, *p* < 0.05). The power ratio was also significantly lower in OA–TOF patients with dysrhythmia compared to those without and significantly lower even in OA–TOF patients without dysrhythmia compared to controls, which is suggestive of impaired gastric contractility in these patients. However, there was no statistical difference between the power ratio of OA–TOF patients with and without dysrhythmia. OA–TOF patients with dysrhythmias also had significantly higher mean spectral frequencies than patients without dysrhythmias in both fasting and postprandial states (*p* < 0.05). There were no differences in mean spectral frequencies between OA–TOF patients without dysrhythmia and controls, unlike the power ratio. However, currently, there is no data in the literature that describes the role of mean spectral frequencies in an EGG study. Most studies use the parameters described above. All five dysrhythmic patients were asymptomatic. A contrast study, which is neither specific nor sensitive for the diagnosis of GORD, was used to evaluate reflux in this study. Contrast study showed GORD in 3/5 (60%) of the dysrhythmic patients. The authors postulated that the dysrhythmia detected in the OA–TOF patients might be due to deficiency of intrinsic inhibitory innervation or a lack of extrinsic autonomic inhibition, a theory supported by prior research by Nakazato et al. and Qi et al.

Gastric myoelectrical activity and gastroesophageal disease were also studied in infants with OA–TOF by Bokay et al. (37). Fifteen OA–TOF infants (mean age of 84 days) and 10 controls were investigated *via* EGG and 24 h oesophageal pH monitoring for the OA–TOF infants. A total of 73.3% of the OA–TOF patients had an abnormal pattern when compared to the controls (10%) during the fasting period. The authors used a cutoff of less than 60% of their percentage of normal slow waves (2–4 cpm) to define an abnormal EGG. In the postprandial phase, a significant increase in bradygastria and a decrease in tachycardia were observed in the OA–TOF cohort. No significant difference was found in the dominant power between the two groups, either before or after the meal. The authors postulated the dysrhythmia seen was due to the abnormalities in Auerbach plexus, leading to poor propagation of electrical potential which in turn results in uncoordinated smooth muscle contraction and peristalsis. They felt that the abnormal gastric electrical activity, during the fasting and/or postprandial period, may lead to uncoordinated contraction of the stomach. Among the 15 OA–TOF patients, 9 had pathological 24 h oesophageal pH monitoring values, and 6 had clinical reflux based on symptoms. When comparing the patients with or without GORD, there were no differences in the distribution of myoelectrical waves or the dominant power, either at rest or after the meal. No information regarding the power ratio was available in this study, making it difficult to compare these results with Yagi et al.’s study. Bokay et al. concluded that EGG is a useful non-invasive investigation to document disturbed neuromuscular function, even in infants, and further studies are required to understand the pathophysiology of feeding disturbances in this population.

To summarize, although EGG is an easy to perform, non-invasive tool to investigate myoelectrical activity, the lack of standardization of the EGG parameters described in the various studies, makes it is difficult to compare the studies. Also, in the literature, the test meal is poorly described. The different test meals in these three studies reflect this statement.

### Dumping Syndrome—OGTT

Dumping Syndrome is thought to occur when a rapid transit of gastric contents reaches the small bowel; resulting in an early postprandial hyperglycemia, which then, leads to a profound insulin response producing a secondary late hypoglycemia. The symptoms can be non-specific and can present with malaise, lethargy, nausea, retching, failure to thrive, diaphoresis, tachycardia, and watery diarrhea. The gold standard for the diagnosis of dumping syndrome is OGTT. Serial blood sugar measurements are done during a 4-h period following a sugar load (1.75 g/kg, maximum 75 g) to detect early hyperglycemia or late hypoglycemia. The treatment of dumping syndrome is mainly by dietary modification by avoiding simple carbohydrates, supplementation with complex carbohydrates (corn starch, pectin), continuous gastric or transpyloric feeds. Some studies have also reported a benefit with Acarbose (38). Rarely octreotide, diazoxide, prednisolone, or even TPN is needed in severe cases. The increased risk of dumping syndrome in adults after oesophageal, gastric, or bariatric surgery is well established (39). Most the studies focus on post-fundoplication (antireflux surgery) dumping syndrome (40). Holschneider et al. (41) investigated the complications following fundoplication in children with a focus on OA–TOF. The incidence of postoperative dumping syndrome has been reported to be significantly higher (18.3%) in the OA–TOF cohort when compared to children without OA–TOF (1.6%). Michaud et al. (42) were the first group to report two cases of symptomatic OA–TOF children without previous fundoplication or associated microgastria who presented with dumping syndrome diagnosed with OGTT. They suggested that dumping syndrome should be considered in OA–TOF children who present with non-specific gastrointestinal symptoms that cannot be explained otherwise (e.g., anastomotic stenosis, gastroesophageal reflux, oesophageal dysmotility, etc.). Large multicentre prospective studies are required to determine the true incidence of dumping syndrome in OA–TOF patients. Although OGTT is the gold standard for the diagnosis of dumping syndrome, the role of complementary GES (showing accelerated GE) remains yet to be determined. In addition, normative values for OGTT have also not been firmly established, especially for all ages for the diagnosis of dumping syndrome. Given these findings, it is important that clinicians consider dumping syndrome in every child treated surgically for oesophageal atresia presenting with digestive symptoms, malaise, failure to thrive, or refusal to eat. Dumping syndrome is often underdiagnosed in this cohort because of the non-specific clinical symptoms and because the GI symptoms are often thought to be due to other more commonly occurring factors, such as strictures, GORD, and dysmotility.

### Symptoms Questionnaires

Gastroesophageal reflux is often diagnosed based on non-specific symptoms, which is not ideal especially for the younger child. The NASPGHAN-ESPGHAN guideline for the diagnosis and treatment of GORD in children states that in infants and toddlers, there is no symptom or symptom complex that is diagnostic of GORD or predicts response to therapy (20). This is especially so in the OA–TOF patient in whom symptoms secondary to eosinophilic oesophagitis, anastomotic stricture, and dysmotility could mimic reflux disease. There is also a dearth of validated questionnaires to evaluate GORD in all age groups and to evaluate and diagnose abnormal gastric function vs. GORD. Hence, it is not surprising that none of the studies mentioned above which evaluated gastric function and its correlation with gastrointestinal symptoms used a validated symptom questionnaire.

Although the PedsQL currently has subsections which relate to gastric dysfunction, further studies are needed to establish more specific validated questionnaire for children and their parents regarding the symptoms related to gastric dysfunction. Significantly, none of the studies on GE, myoelectrical activity, and motility found a significant correlation between the abnormalities in gastric function and symptoms. This lack of correlation might well be due to not only the small sample sizes but also the lack of a validated symptom questionnaire for gastric function.

## Treatment of Abnormalities in Gastric Function

### Role of Prokinetic

The studies investigating the use of prokinetic in OA–TOF patients are scarce. Most of them evaluate the benefits of prokinetics in OA–TOF patients with oesophageal dysmotility or GORD. No study so far has specifically looked at the role of a prokinetic on gastric function in OA–TOF patients. Ideally, the effectiveness of the prokinetic drug should be evaluated not only on pathophysiologic changes in EGG and GE but also on patient-related outcomes with validated symptom questionnaires. Prokinetic drugs can improve gastric motor function/emptying by accelerating rate of GE, and by their effect on gastric peristalsis.

Cisapride increases the motility of the upper gastrointestinal tract by acting directly as a serotonin 5-HT_4_ agonist and indirectly as a parasympathomimetic. Its action on the serotonin receptors increases the release of acetylcholine in the enteric nervous system, improving the GE. Tegaserod has a similar mechanism by being a 5-HT_4_ receptor agonist. However, both cisapride and tegaserod presented significant side effects, mainly cardiac, leading to a withdrawl of their use (43, 44).

Domperidone is a peripheral dopamine antagonist with affinity for D2-receptors that increases motility and GE (45). It works by antagonizing the effects of dopamine on the gastrointestinal tract, but has no cholinergic activity. It does this by inhibiting fundal relaxation, and by increasing amplitude and peristalsis of the gastric antrum and duodenum. Studies show mixed results regarding symptomatic improvements. Its efficacy was mainly investigated in diabetes gastroparesis, with reduction in nausea and vomiting. However, the trials were small and had methodological limitations (46). Domperidone has been shown to improve gastric dysrhythmia in diabetic gastroparesis (47, 48). Three aspects should be considered when prescribing domperidone. First, it has the propensity to increase the QT interval on electrocardiogram, potentially leading to arrhythmia. Therefore, baseline and follow-up electrocardiogram are recommended, and Domperidone should be discontinued in case of age-related prolonged corrected QT interval (49). Second, domperidone increases prolactin and can result in mild lactation. Third, it alters the function of the cytochrome P450 2D6, theoretically increasing the risk of drug interaction.

Erythromycin, in a sub-antimicrobial dose, has been used in gastroparesis. It mimics the effect of motilin in the proximal gastrointestinal tract, provoking migrating motor complexes and contractions in the antrum and duodenum *via* cholinergic actions (50, 51). Several studies have documented an accelerated GE both in healthy controls and with patients with gastroparesis (52, 53). However, the results were not consistent with some studies showing a poor response (54). Erythromycin also leads to downregulation of the motilin receptor, inducing a tachyphylaxis. Some studies documented a drop of the response after 4 weeks of treatment (55). As with domperidone, erythromycin interacts with other drugs metabolized by the cytochrome P450 3A4. Like Domperidone, it can also be associated with the development of prolonged corrected QT interval, which necessitates close monitoring during its use.

### Role of Gastric Pacing

Gastric pacing, or gastric electrical stimulation, is a surgical treatment option. It has been evaluated in patients with refractory gastroparesis (56). After the placement of the electrodes into the muscle layer of the stomach, several modalities of stimulation are available, of which, high-frequency/low-energy stimulation with short pulse stimulation is the one most often described. There are currently no studies that have evaluated the effect of gastric pacing in the OA–TOF cohort. However, there might potentially be a role for gastric pacing in OA–TOF patients with significant feeding difficulties and vomiting not responding to conventional therapy who have documented abnormalities in GE and myoelectrical activity.

## Conclusion

Due to the substantial reduction of mortality in patients with oesophageal atresia and TOF, the aim of clinicians looking after OA–TOF patients has shifted to improvements of patient-related outcomes and reduction of the morbidity of gastrointestinal disease affecting them. In the past, the literature has focused on GORD, oesophageal dysmotility, and feeding difficulties. However, the evidence that abnormalities in gastric function can contribute to symptoms such as vomiting, dyspepsia, and feeding difficulties is increasing. This review provides an overview of the pathophysiology of abnormal gastric function in this cohort, and the armamentarium of investigations available to gastroenterologists to diagnose abnormal gastric function. The standardization of the methods, especially the test meals and the establishment of rigorous standards, are mandatory to determine normal values for GE and EGG in children.

Even with limited literature currently available on this topic, this review highlights the importance of being aware of the risk of gastric dysfunction in oesophageal atresia and TOF patients. We have described the investigation of gastric function with objective tests, such as GES or OBT to evaluate GE, EGG to evaluate gastric myoelectrical activity, and OGTT to exclude dumping syndrome. Potential treatment modalities for these abnormalities in gastric function have also been described.

Although most of the studies described had small cohorts, they all showed abnormalities in GE and myoelectrical activity in a significant proportion of OA–TOF patients. However, none of the studies could conclusively show a significant correlation between the abnormalities in gastric function and symptoms, although that might well have been due to small sample sizes and lack of a specific validated symptom questionnaire.

## Future Directions

Several countries have launched a national plan for rare diseases, thus, increasing the awareness of conditions, such as OA–TOF, such as NORD in the United States, or EURORDIS in Europe. Recently, members of the European and North American Society of Pediatric Gastroenterology, Hepatology and Nutrition developed uniform consensus guidelines for the management of gastrointestinal complications in children with OA–TOF (3). This illustrates the need for collaborations in the field of rare diseases. To improve our understanding of gastric function in OA–TOF, multicentre collaborative prospective trials are needed. Only such large multicentre studies will help determine whether treating abnormalities in GE and myoelectrical activity improves GORD, dyspepsia and feeding difficulties in OA–TOF patients.

Patient-related outcome instruments, including the development of validated patient symptom, and parent-proxy questionnaires are essential in the development of treatment modalities, assuring therapeutics benefits to the patients.

## Author Contributions

GD reviewed the current literature, elaborated a plan for the review, and wrote the manuscript. UK provided additional help for the review and wrote and corrected the manuscript.

## Conflict of Interest Statement

The authors declare that the research was conducted in the absence of any commercial or financial relationships that could be construed as a potential conflict of interest.
